# Case Report: Delayed Primary Wound Closure After Extensive Abdominal Wall Resection for Infection and Malignancy Using TopClosure®

**DOI:** 10.3389/fsurg.2021.684513

**Published:** 2021-05-20

**Authors:** Evgeny Solomonov, Muhammad Khalifa, Vladimir Rozentsvaig, Itzhak Koifman, Seema Biswas, Moris Topaz

**Affiliations:** ^1^Ziv Medical Center, Safed, Israel; ^2^Galilee Medical Center, Nahariya, Israel; ^3^IVT Medical Ltd., Ra'anana, Israel

**Keywords:** open abdomen, top closure, damage control, abdominal wall, massive abdominal wall defect

## Abstract

The closure of a massive abdominal wall defect is illustrated using a novel dynamic closure technique - the TopClosure® tension relief system. This system attaches to the abdominal wall immediately after laparotomy and allows for early approximation of the skin, avoiding an open abdomen and the complications associated with this. The technique in this case was employed after extensive resection of the abdominal wall for infected skin metastases of colonic adenocarcinoma and circumvented post-operative ventilation and open abdomen. Early recovery after such extensive surgery is important in terms of patient morbidity and mortality. In this case, primary surgery may not have been an acceptable risk to undertake without the option of Top Closure of the abdomen. We illustrate the technique of abdominal wall closure through a series of images of the procedure.

A 41-year-old woman with recurrent, infected, ulcerated colonic adenocarcinoma and extensive involvement of the abdominal wall underwent resection of the sigmoid colon, radical right iliac lymph node dissection and substantial excision of the abdominal wall (Figures 1A,B). On induction and for the first post-operative 24 h she received standard hospital antibiotic prophylaxis – 1g amikacin and 500 mg metronidazole intravenously. Vicryl mesh was sutured to the margins of a 490 cm^2^ defect (bordered by the right linea alba, residual right rectus abdominis muscle, right subcostal abdominal wall, right flank along the length of the right iliac bone from the iliac crest to the pubis, and laterally from the remnant of the right external oblique muscle and anterior superior iliac spine to the fascia lata of the right leg) to cover the small and large bowel (Figure 1C).

**Figure 1:**
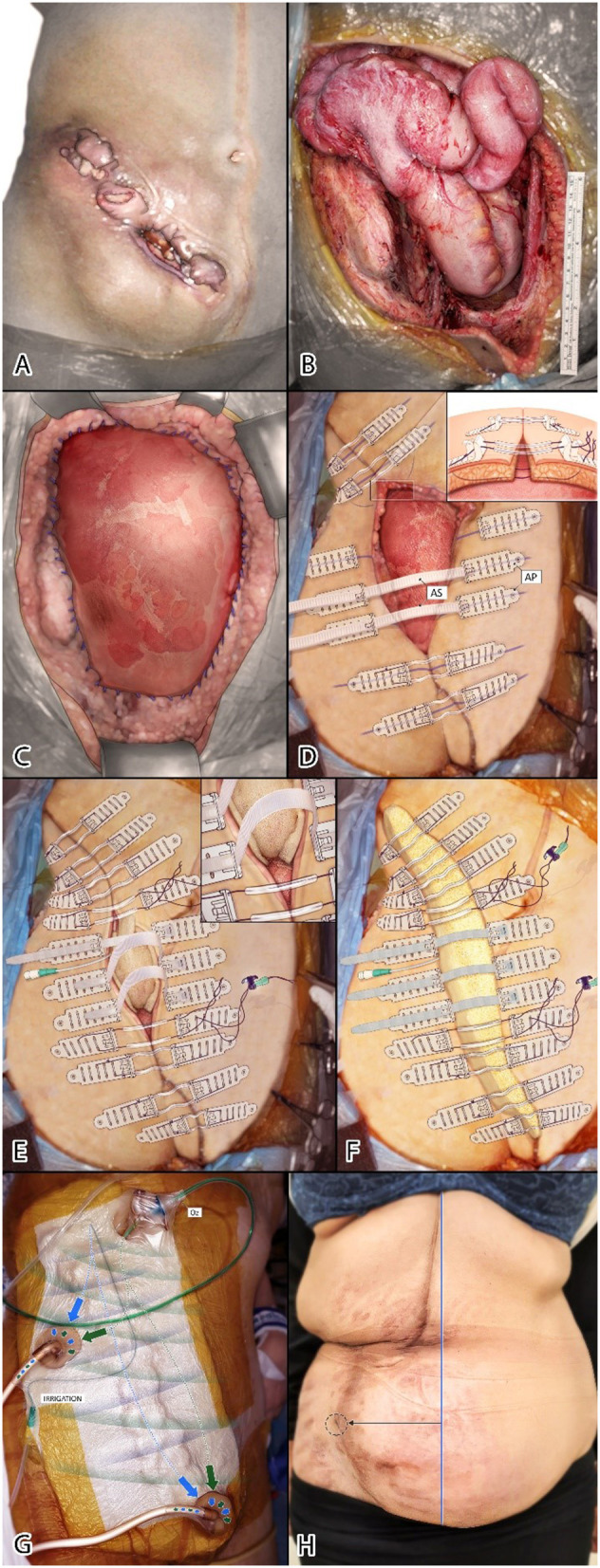
**(A)** Recurrent ulcerated colonic adenocarcinoma involving abdominal wall. **(B)** Residual 26.5 × 18.5 cm abdominal wall defect. **(C)** Vicryl mesh sutured to margins of defect. **(D)** TopClosure sets applied perpendicular to wound, 3–4 cm from wound edges (insert shows relation of tension sutures to AP and underlying structures). **(E)** First stage wound approximation (insert; inner layer fenestrated non-permeable surface of sponge apposing Vicryl mesh). **(F)** Second sponge layer under ASs. ASs in center of wound permit further approximation by mechanical creep. **(G)** Complete cover and insulation of wound by ROI-NPT apparatus. **(H)** Six months after surgery. Lateral deviation (black line) from midline (blue line) of the umbilicus (circle).

The left abdominal wall was stretched across to the right using 1/0 nylon tension sutures running over nine of twelve TopClosure® attachment plates (AP) (IVT Medical Ltd, Ra'anna, Israel) (Figure 1D–insert). Approximation of ~95% of the wound was achieved by stress relaxation within 85 min, leaving a central 3.5 × 8 cm (28 cm^2^) defect, bridged by three APs to be pulled together gradually postoperatively by their connecting approximation straps (AS) (Figure 1D). A soft, compressible open-cell sponge, a component of the Vcare α^®^ system (IVT Medical Ltd., Ra'anna, Israel) was placed in the defect–the impermeable polyurethane layer in apposition with the Vicryl mesh–pushing down to obliterate a potential dead-space and protect the bowel from direct contact with the sponge to avoid fistulation (Figures 1E,F). A few slits (fenestrations) were cut into the impermeable layer to permit evacuation of intraperitoneal fluid. An irrigation catheter for continuous *in-situ* ultra-high dose antibiotic (CITA) (160 mg gentamicin in 100 ml saline/24 h) was placed over the Vicryl mesh and directed toward the costal margin to promote dispersion of antibiotic solution throughout the wound cavity. Regulated oxygen and irrigation–negative pressure-assisted therapy (ROI-NPT) tubes were sited at opposite limits of the wound to create unidirectional, three-dimensional flow (Figure 1G).

A low-pressure vacuum was applied using the Vcare α^®^ set to 50 mmHg (oscillating between 40 and 60 mmHg) to reduce the risk of bleeding and fistulation. The primary function of the vacuum in this application was to decontaminate the wound and evacuate fluid rather than promote angiogenesis and granulation. With the wound dressed, slow skin stretching by mechanical creep was performed daily by tightening the AS at the bedside without the need for frequent dressing changes. Serum gentamicin levels were checked routinely and remained within the normal range.

Formal dressing changes and staged closure of the residual gap were performed in the operating room on the 5th and 9th post-operative day (when complete closure was achieved).

The ROI-NPT was removed on the 17th post-operative day and the patient was discharged home the next day with instructions to keep the wound and APs clean. Dressing changes and formal wound care were not required at home.

At 6 weeks follow up, a central discharging seroma was evident at the lateral aspect of the original defect adjacent to the right iliac crest (where there was no adjoining/overlying tissue). The wound was partially reopened, irrigated, and a sponge cylinder inserted with an antibiotic irrigation catheter as before. Two new AP sets were applied across the defect to close the wound in stages, and ROI-NPT reinstituted for another 2 weeks until complete closure.

Six months after surgery, the wound is entirely healed. The umbilicus has been pulled laterally into the right iliac fossa (Figure 1H).

TopClosure® reduces shear stress by means of its wide area of attachment to the skin. Dynamic stretching of the skin is through both repeated low-tension mechanical creep and fast high-tension stress relaxation using deep tension sutures (1). The APs serve as a tension-relief platform for tension sutures when high tension is indicated to avoid damage to the underlying skin (2, 3). Skin ischemia is substantially reduced by placing the APs on intact skin (non-traumatized and non-undermined) 3–4 cm from the wound edges and by avoidance of any undermining of the skin (unnecessary as the tension relief system already pulls the skin edges together, and discouraged so that blood supply to the skin is optimal and any new potential space for infection or fluid collection is obliterated).

The Vcare α^®^ is an advanced vacuum therapy system which may be combined with simultaneous regulated oxygen and irrigation with safety features. These safety features include software that requires the operator to define the risk of bleeding (high, medium, or low) and, as a result, select a program with pre-determined vacuum pressure settings and corresponding alarm features. Sensors in the Vcare α^®^ system detect filling of the Vcare α^®^ canister. When specified rates are exceeded (as in the case of bleeding), audio and visual alarms and automatic cessation of the vacuum are triggered. This feature is augmented by the use of a semi-transparent (off-white) sponge that permits easy visualization of bleeding (and infection). Supplemental oxygen reverses the reduced partial pressure of oxygen in the wound induced by conventional vacuum systems, and may inhibit anaerobic bacterial growth (4, 5). Concurrent antibiotic irrigation of the wound hydrodynamically accelerates the evacuation of infectious material from the wound (6) and may effectively decontaminate the wound (7).

In this patient, simplifying primary wound closure avoided the morbidity of an open abdomen and hastened recovery. The patient did not require postoperative ventilation—avoiding associated pulmonary and circulatory complications. Nasogastric feeding was commenced immediately after surgery. Recovery was pain-free and thus, early mobilization and return to normal bowel function were facilitated. Obliteration of any residual wound cavity and complications of seroma were dealt with using the same apparatus and technique. This relatively simple procedure does not require advanced operating theater apparatus.

The application of TopClosure® may circumvent the open abdomen in patients left with massive abdominal wall defects at the end of laparotomy by dynamic stretching. Thus, complications associated with prolonged intubation and ventilation, fluid balance, the catabolic state, wound infection, abdominal fluid and protein loss, further loss of abdominal wall domain, and development of enteroatmospheric fistula are avoided (8, 9). Local rotational flaps, free flaps, such as latissimus dorsi or fascia lata flaps (10), are surgical alternatives; but, surgical complexity, donor site morbidity, and ischemic or septic flap failure may be prohibitive factors in opting for flap reconstruction. Complete or near complete approximation of wounds at first-stage surgery renders TRS and ROI-NPT a damage control technique that permits extensive surgery in patients with advanced malignancy, infection, or significant potential morbidity who might otherwise be advised against surgical intervention.

## Data Availability Statement

The original contributions presented in the study are included in the article/supplementary material, further inquiries can be directed to the corresponding author/s.

## Ethics Statement

Ethical review and approval was not required for the study on human participants in accordance with the local legislation and institutional requirements. The patient provided written consent for all parts of surgery. Written informed consent was obtained from the individual(s) for the publication of any potentially identifiable images or data included in this article.

## Author Contributions

ES, SB, and MT wrote the manuscript. All authors contributed to the article and approved the submitted version.

## Conflict of Interest

MT is inventor of the TopClosure^®^, Vcare α^®^, and Mecare α^®^ systems, and CEO of IVT Medical Ltd that manufactures the devices. SB is clinical researcher at IVT Medical Ltd. The remaining authors declare that the research was conducted in the absence of any commercial or financial relationships that could be construed as a potential conflict of interest.

## References

[1] Topaz M Carmel NN Topaz G Li M Li YZ. Stress-relaxation and tension relief system for immediate primary closure of large and huge soft tissue defects: an old-new concept. Medicine. (2014) 93:e234. 10.1097/MD.0000000000000234 25526444 PMC4603089

[2] Topaz M Carmel NN Silberman A Li MS Li YZ. The TopClosure® 3S system, for skin stretching and a secure wound closure. Eur J Plast Surg. (2012) 35:533–43. 10.1007/s00238-011-0671-1 22719176 PMC3375424

[3] Katzengold R Topaz M Gefen A. Tissue loads applied by a novel medical device for closing large wounds. J Tissue Viability. (2016) 25:32-40. 10.1016/j.jtv.2015.12.003 26750452

[4] Topaz M Bisker O Litmanovitch M Keren G. Application of regulated oxygen-enriched negative pressure-assisted wound therapy in combating anaerobic infections. Euro J Plast Surg. (2011) 34:351–58. 10.1007/s00238-010-0514-5

[5] Li YZ Hu XD Lai XM Li YF Lei Y. Improvement of wound healing by regulated oxygen-enriched negative pressure-assisted wound therapy in a rabbit model. Clin Experim Dermatol. (2018) 43:11–8. 10.1111/ced.13225 28940698

[6] Motiei M Sadan T Zilony N Topaz G Popovtzer R Topaz M. Gold nanoparticles for tracking bacteria clearance by regulated irrigation and negative pressure-assisted wound therapy. Nanomedicine. (2018) 13:1835–945. 10.2217/nnm-2018-0053 30152260

[7] Topaz M Kazatsker M Ashkenazi I Schwartz AL Carmel-Neiderman NN Topaz G. A novel antibiotic delivery approach enhances salvage of infected cardiovascular-implantable electronic devices. Circulation. (2020) 142:A15682. 10.1161/circ.142.suppl_3.15682

[8] Santivañez Palomino JJ Vergara A Cadena M. Open abdomen: the surgeons' challenge. In Dogan KH editor. Wound Healing – Current Perspectives. London: IntechOpen (2019). p. 1–14. 10.5772/intechopen.81428

[9] Coccolini F Biffl W Catena F Ceresoli M Chiara O Cimbanassi S. The open abdomen, indications, management and definitive closure. World J Emerge Surg. (2015) 10:32. 10.1186/s13017-015-0026-5 26213565 PMC4515003

[10] Mericli AF Baumann DP Butler CE. Reconstruction of the abdominal wall after oncologic resection: defect classification and management strategies. Plast Reconstr Surg. (2018) 142:187–96S. 10.1097/PRS.0000000000004877 30138289

